# Supplementary material for: Empirical methods for controlling false positives and estimating confidence in ChIP-Seq peaks
Source: BMC Bioinformatics. 2008 Dec 5;9:523. doi: 10.1186/1471-2105-9-523 (PMC2628906; doi:10.1186/1471-2105-9-523)
Supplement: Additional File 1 — A variety of html documents from the USeq web site detailing the available applications, their best usage, output file type descriptions, command line menus, etc. [file 1471-2105-9-523-S1.zip › USeqUserGuides/JavaDoc.zip!/JavaDoc/trans/graphics/class-use/CelMasker.html]

Uses of Class trans.graphics.CelMasker


|  |  |  |
| --- | --- | --- |
| **Overview** \| **Package** \| **Class** \| **Use** \| **Tree** \| **Deprecated** \| **Index** \| **Help** | |  |
| PREV   NEXT | **FRAMES**    **NO FRAMES**     **All Classes** |  |


---


## **Uses of Class trans.graphics.CelMasker**

| Packages that use CelMasker | |
| --- | --- |
| **trans.graphics** | For graphically representing '.cel' files and intervals. |

Uses of CelMasker in trans.graphics

| Methods in trans.graphics that return CelMasker | |
| --- | --- |
| `CelMasker` | `VirtualCelFrame.getCelMasker()` |

| Methods in trans.graphics with parameters of type CelMasker | |
| --- | --- |
| `void` | `VirtualCelFrame.setCelMasker(CelMasker celMasker)` |

Constructors in trans.graphics with parameters of type CelMasker

`VirtualCel(float[][] intensityValues,
CelMasker celMasker)`
  
          For integration with CelMasker.

---


|  |  |  |
| --- | --- | --- |
| **Overview** \| **Package** \| **Class** \| **Use** \| **Tree** \| **Deprecated** \| **Index** \| **Help** | |  |
| PREV   NEXT | **FRAMES**    **NO FRAMES**     **All Classes** |  |


---
